# Supplementary material for: The effect of intrinsic crumpling on the mechanics of free-standing graphene
Source: Nat Commun. 2015 Nov 6;6:8789. doi: 10.1038/ncomms9789 (PMC4667622; doi:10.1038/ncomms9789)
Supplement: Supplementary Information — Supplementary Figures 1-7 and Supplementary References [file ncomms9789-s1.pdf]

Supplementary Figures

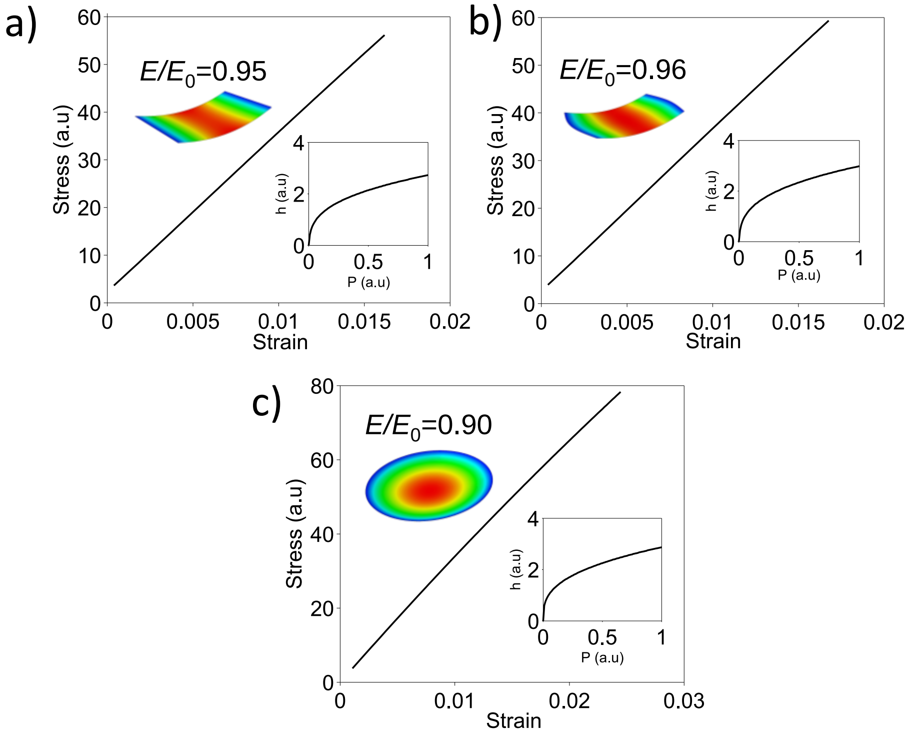

Supplementary Figure 1: Finite element analysis of loaded membranes with various geometries Finite-element modelling (FEM, ABAQUS 6.14) was used to confirm accuracy of the analytical formulas (Eq.1) used in the main text to extract stress and strain from pressure and center-point deflection of graphene membranes. We modeled the same geometries used in our experiments: a ribbon *(a)*, a ribbon with curved ends *(b)*, and a circular membrane *(c)*. Shell elements with fixed edges were used, the stiffness of membranes was set to an arbitrary $E_0 = 3.5$ GPa. We then used computationally obtained center-point deflection vs. pressure dependence (insets) to find stress and strain (main panels) and extracted the stiffness E using the same analytic formulas as in the main text. In all cases, we found that the obtained E does not deviate from the pre-set E_0 by more than 10%.

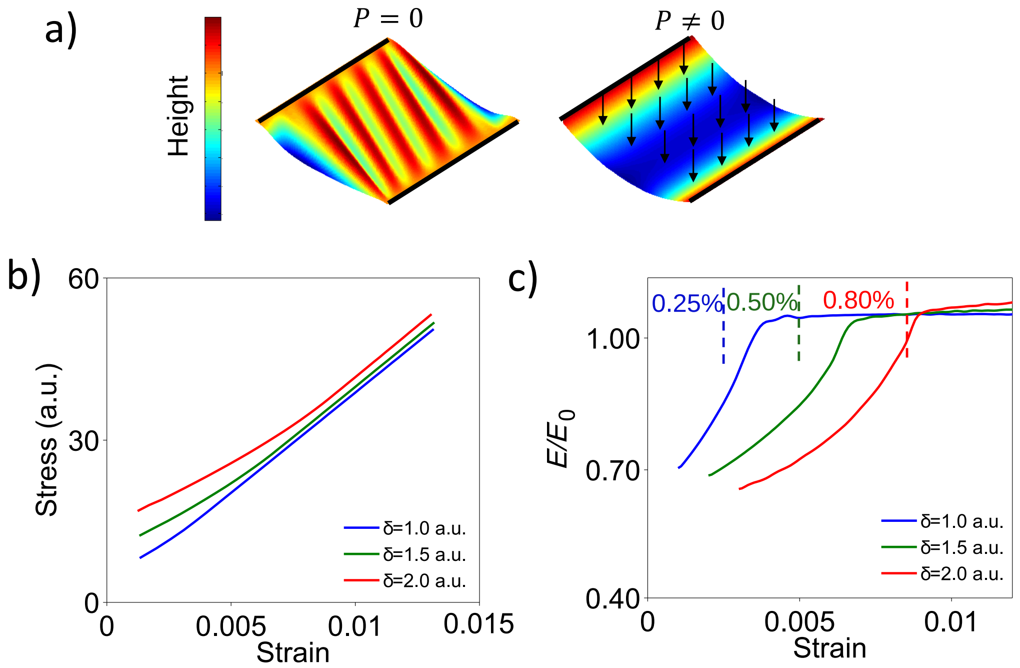

Supplementary Figure 2: Finite element modeling of wrinkled membranes

We used FEM to confirm that wrinkling leads to softening of the effective in-plane stiffness and produces non-linear stress-strain curves (Fig. 2 of the main text). We used a rectangular ribbon geometry with wrinkles induced following the approach by Wong et al.[1] The membrane was loaded under constant pressure *(a)*. Its effective stress and strain *(b)* were determined using the same analysis as in Supplementary Figure 1 and the main text. We observed a non-linear stress-strain relationship with the degree of non-linearity depending on wrinkle amplitude δ. The effective stiffness of wrinkled membranes was calculated as a derivative of the stress-strain curve normalized by the stiffness of unwrinkled membrane E_0. *(c)* The membrane is seen softened in the regime of small strain.

We also used the FEM data to confirm the conjecture used in the manuscript, that graphene appears softened below a threshold strain $\varepsilon_\mathrm{t} = \Delta A/2A = (A' - A)/2A$ (A is the surface area of a membrane, A' is the area of the projection of a membrane onto a plane parallel to it). The positions corresponding to threshold strains for different amounts of wrinkling are shown by dashed lines in *(c)*. We indeed find that the $E < E_0$ below ε_t, and that $E \sim E_0$ above ε_t.

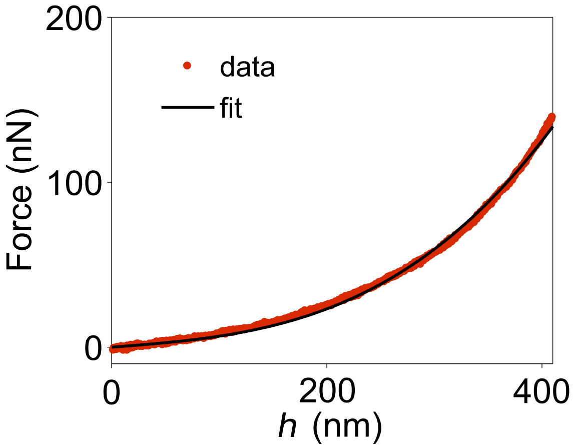

Supplementary Figure 3: Atomic force microscopy indentation

We carried out AFM nanoidentation measurements on our devices to cross-check our experimental results. We followed the approaches of Lee et al.[2] and Ruiz-Vargas et al.[3] Bruker AFM (Dimension Icon) with Budget Sensors Multi75Al cantilevers were used. Prior to measurement, the cantilever spring constant ($k{\sim}3$ N/m) was measured via thermal tuning and the deflection sensitivity was obtained by pushing the cantilever against a hard substrate (silicon). Suspended graphene membranes (diameter 10 μm) were imaged in tapping mode to determine their center and indented in contact mode to measure ramp distance z and cantilever deflection δ. The resulting force (F) – indentation (h) curves (red points) were extracted as: $F = -k\delta$ and $h = z - \delta$. Then, the in-plane stiffness $E_{2\mathrm{D}}$ and built-in stress σ_0 were determined by fitting $F(h)$ curve (black) to the nanoindentation equation:

$$F = \sigma_0 \pi h + E_{2D} q^3 \frac{h^3}{a^2} \qquad (1)$$

Here a is membrane radius, constant q = 1.02 is related to the Poisson's ratio of graphene. We note that our analysis does not change significantly if we include the radius of the AFM tip as used in some other models.[3] From the fit, we obtain $E_{2\mathrm{D}} = 29 \pm 1$ N/m, similar to the result obtained from interferometry profilometry. For circular perforated membranes, we found much higher stiffness $E_{2\mathrm{D}} = 199 \pm 5$ N/m, once again in qualitative agreement with our results from interferometric profilometry. We note that nanoidentation can only be reliably applied to circular membranes, and not to ribbons.

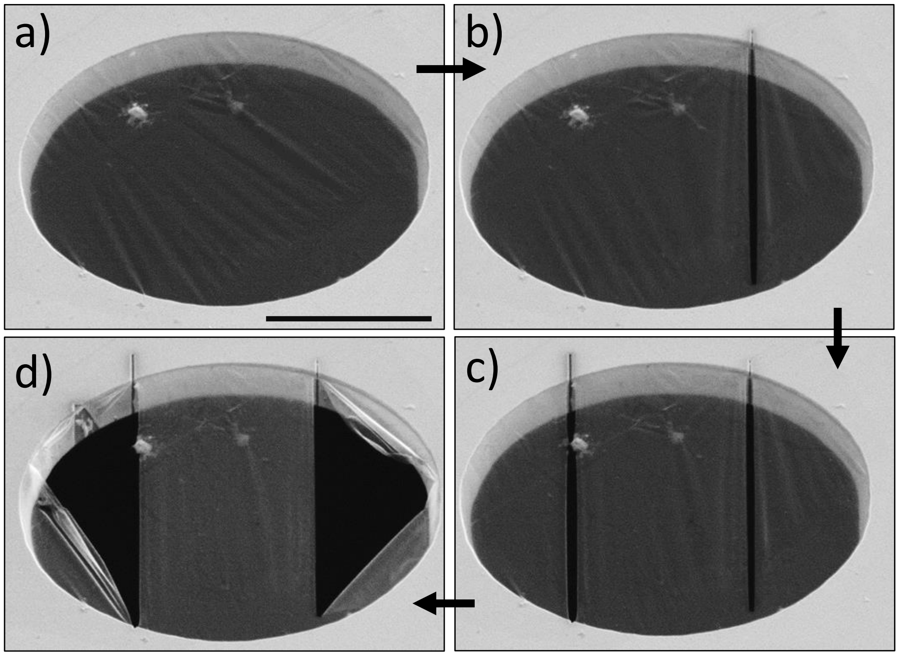

Supplementary Figure 4: The effect of FIB cutting on wrinkles
Wrinkles in graphene membranes are suppressed by the cutting procedure. We observed that successive cuts by focused ion beam (a,b,c,d) reduce the amplitude of the wrinkles and reorient them along the cuts. We note that this is an unusually wrinkled sample chosen for illustration. Most samples exhibited no visible wrinkles under similar imaging conditions. The scale bar is 5 μm.

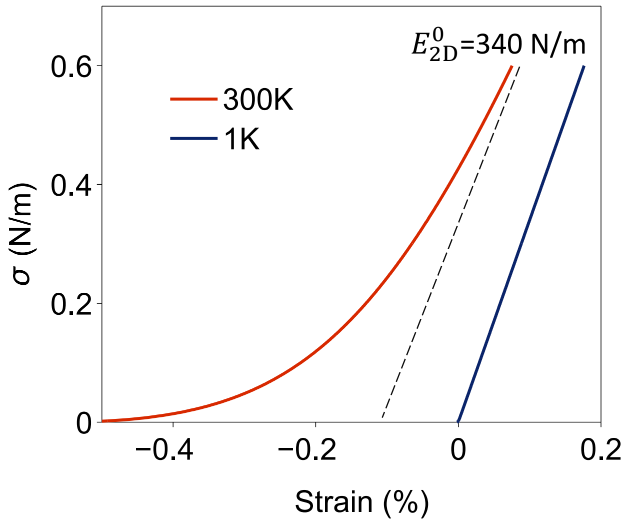

Supplementary Figure 5: Analytical description of flexural phonons in free-standing graphene
To obtain an approximate analytical expression for stress-strain dependence of a membrane perturbed by flexural phonons, we built upon a model originally developed for lipid membranes.[4] From the equipartition theorem, we find the average amplitude u_q of a flexural phonon with wavevector q:

$$\langle |u_q|^2 \rangle = \frac{k_B T}{A(q^4\kappa(q) + q^2\sigma)} \qquad (2)$$

Here k_B is Boltzmann constant, T is temperature, σ is externally applied stress, and κ is membrane's bending rigidity. While κ is constant for all the modes for the lipid membranes considered in Ref. 4, the interplay between bending and stretching makes κ wavevector-dependent in graphene. Following recent literature, we approximate $\kappa(q) = \kappa_0 + k_B T B (q_0/q)^\eta$, with B= $5.9T^{(\eta/2-1)}$, $q_0 = 2\pi(E^0_{2D}/\kappa_0)^{1/2}$, $\eta = 0.85$, $E^0_{2D} = 340$ N/m and $\kappa_0 = 1$ eV.[5-9]

Next, we calculate the amount of crumpling, as defined in the main text, due to a single flexural phonon mode:

$$\left(\frac{\Delta A}{A}\right)_q = q^2 |u_q|^2 \qquad (3)$$

The total amount of crumpling in a membrane is found by integrating over all available flexural phonon modes:

$$\frac{\Delta A}{A} = -\frac{1}{2\pi}\int_{q_{min}}^{q_{max}} \frac{k_B T}{(q^2\kappa(q) + \sigma)} q\, dq \qquad (4)$$

In this expression the lower integration limit $q_{min} = 2\pi/\sqrt{A}$ is related to membrane's size, while the upper limit $q_{max} = \sqrt{3kTE^0_{2D}/8\kappa_0^2\pi}$ roughly speaking only counts phonons that contribute to stiffness.[9] Finally, we compute the amount of strain in a crumpled membrane as:

$$\varepsilon = \frac{1}{2}\frac{\Delta A}{A} + \frac{\sigma}{E^0_{2D}} \qquad (5)$$

The two terms in this expression are stemming from two sources of strain – crumpling due to flexural phonons (first term) and stretching of carbon/carbon bonds with effective stiffness $E_{\mathrm{2D}}^{0} = 340N/m$.

The stress-strain curves obtained numerically from this last equation for different temperatures are shown in Fig. 5. At $T = 1$ K, the flexural phonons are almost completely suppressed and the stiffness of graphene is close to $340\ \mathrm{N/m}$. At $T = 300$ K, graphene is wrinkled and appears softer. To fit our experimental data (Fig. 3) to Supplementary Equation 5, we took $E_{\mathrm{2D}}^{0} = 85N/m$, our measured stiffness at $T = 10$ K.

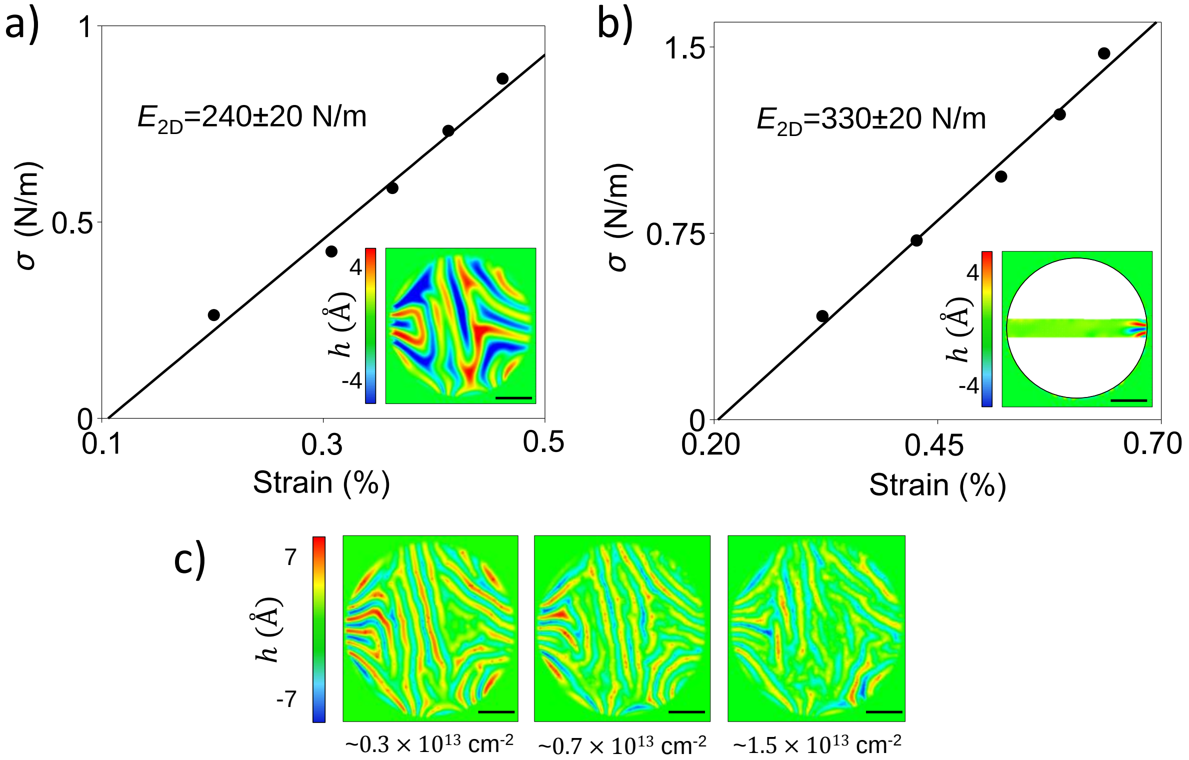

Supplementary Figure 6: Molecular Dynamics modeling of wrinkles in graphene membranes, cut graphene membranes, and graphene membranes with defects

We used Molecular Dynamics (MD) modeling to confirm that cutting circular membranes into ribbons suppresses wrinkles and stiffens graphene. Classical molecular dynamics (MD) simulations were performed using the LAMMPS package.[10] A bond-order potential[11] was used to model the atomic interactions. First, we only considered the effects due to static wrinkles and not due to flexural phonons by keeping temperature near zero, at $T = 0.1$ K. Left and bottom boundaries along x and y directions were divided into regions, where the interatomic distances were increased or decreased, creating non-uniform strain in the membrane. This non-uniform strain induced wrinkles in the membrane, as shown in Inset a). We used circular boundary conditions similar to the experimental setup. Atoms at the boundary were kept from moving, while the dynamics of the free-to-move atoms in a circular region with the diameter of 92.7 nm was simulated using NPT (constant temperature and pressure) ensemble. To represent the pressure on the membrane, an additional constant force perpendicular to the graphene plane, along the z direction, was applied to the atoms located in the circular region. This force was set to be in the range of values from 10^{-5} to 10^{-4} eV/Å, keeping the membrane in the deformation regime controlled by wrinkles. During the dynamical motion of the membrane we reduced the simulation time, required to reach equilibrium by reducing kinetic energy with a damping force $F = \gamma v$, where $\gamma = 5 \times 10^{-4}$ eV fs Å^{-2} for the circular membrane and where $\gamma = 7.5 \times 10^{-4}$ eV fs Å^{-2} for the cut membrane. The MD system was equilibrated for 100 ps for all pressures and boundary conditions.

We computationally determined stress, strain and stiffness for a wrinkled circular membrane (a) with 384000 atoms for entire 100×100 nm sheet (Inset a) and the same membrane cut into a narrow (100×15 nm ribbon inset b) with 62000 atoms (scale bars 20nm). Consistent with the results described in the manuscript, we found that graphene is softened to ~ 240 N/m by the presence of wrinkles, and that the wrinkles are suppressed and stiffness recovers almost up to 340 N/m after cutting. While the qualitative agreement is adequate, we note that MD results cannot be quantitatively compared to our experimental

data as computational complexity of a problem limits the size of membranes used in simulation to less than ~ 100 nm.

We also used MD simulation to model flexural phonons in graphene. At room temperature graphene undergoes thermal crumpling due to the out-of-plane displacements of atoms. The hidden area ΔA is defined as the difference between the area of the projection on x-y plane and the total area of the graphene sheet. Atomic positions were calculated with molecular dynamics after the system reached thermal equilibrium at room temperature, $T = 300$ K, used in the experiment. We determined the projected area as the product of dimensions of the sheet in x-y directions $A = L_x \times L_y$. Total area is the taken as the area of a flat sheet of graphene with interatomic distances at $T = 300$ K. The ratio of the hidden area to the total area is calculated as an average for 10 snapshots at the thermal equilibrium and found to be $\Delta A/A = 0.0042$.

Finally, we examined the evolution of wrinkle topography upon introduction of defects into graphene lattice. To do this, atoms at random locations were removed to create the desired concentration of defects. Then, the MD simulations identical to those in *(a)* were carried out. The topography of wrinkles for different defect densities 0.3, 0.7 and 1.5×10^{13}cm^{-2} is shown in *(c)* (scale bars 20 nm). The key observation here is that even at moderate defect concentrations, static wrinkles persist in the membranes.

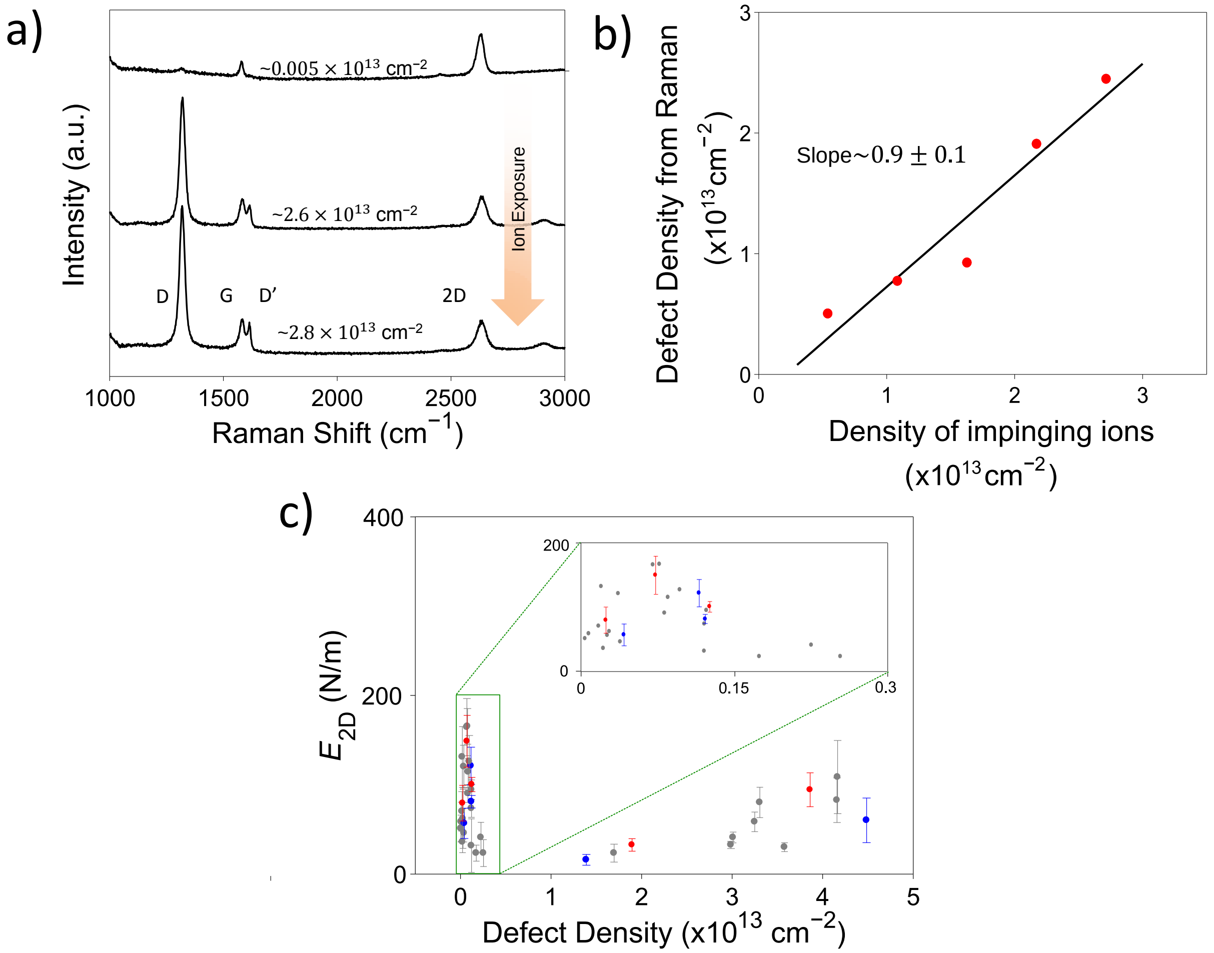

Supplementary Figure 7: The influence of defects upon mechanical response of graphene membranes

We examined changes in the mechanical response of graphene membranes upon introduction of vacancy defects in graphene lattice. To controllably introduce defects, we placed our membranes into an FIB chamber and rastered a 5 keV beam of Ga^+ ions (29 pA) over an area larger than our samples ($100 \times 100\ \mu m^2$). Typical exposure times between 0 and 30s translates into ion doses between $0 - 5 \times 10^{13} cm^{-2}$. To determine the concentration and type of defects upon irradiation, we collected Raman spectra of our devices following the exposure, an example is shown in *(a)* where each spectrum is offset for clarity. The increase in the intensity of the defect-related Raman *D* peak indicates creation of defects. To determine the defect type, we monitored the ratio between *D* and *D'* Raman peaks and determined $I(D)/I(D') \sim 6 \pm 1$ on average across all our samples. This is very close to $I(D)/I(D') = 7$ expected for vacancies.[12] To quantitatively determine the defect concentration, we analyzed the ratio between the *D* and *G* Raman peaks.[13] The results of this analysis, plotted *vs.* the density of ions that impinged onto graphene are shown in *(b)*. The linear fit to this data, with slope ~0.9, suggests that Ga ions produce vacancy-type defects in graphene with ~90% probability, as expected.[14] Finally, we examined the evolution of the in-plane stiffness E_{2D} (measured at room temperature, following Ga+ irradiation) *vs.* defect concentration (as determined from Raman spectra) in the range of defect densities $0 - 5 \times 10^{13} cm^{-2}$. The data shown in panel *(c)* were collected for 14 different devices; two distinct representative devices are shown as red and blue points respectively, the data from the other devices are gray. The error bars are obtained by estimating the standard deviation of E_{2D} measurements. The inset of *(c)* shows a zoomed-in region of low

defect densities. We note that throughout the range of defect concentrations the in-plane stiffness remained <340 N/m. Moreover, no clear trend of E_{2D} *vs.* defect concentration was observed.

Supplementary References

1 Wong, Y. W. Wrinkled membranes Part III: numerical simulations. *Journal of Mechanics of Materials and Structures* **1**, 63-95, doi:10.2140/jomms.2006.1.63 (2006).

2 Lee, C., Wei, X., Kysar, J. W. & Hone, J. Measurement of the Elastic Properties and Intrinsic Strength of Monolayer Graphene. *Science* **321**, 385-388, doi:10.1126/science.1157996 (2008).

3 Ruiz-Vargas, C. S. *et al.* Softened Elastic Response and Unzipping in Chemical Vapor Deposition Graphene Membranes. *Nano Lett.* **11**, 2259-2263, doi:10.1021/nl200429f (2011).

4 Helfrich, W. & Servuss, R. M. Undulations, steric interaction and cohesion of fluid membranes. *Il Nuovo Cimento D* **3**, 137-151, doi:10.1007/BF02452208 (1984).

5 Katsnelson, M. I. *Graphene: Carbon in Two Dimensions*. (University Press, Cambridge, 2012).

6 Nelson, D., Piran, T. & Weinberg, S. *Statistical Mechanics of Membranes and Surfaces 2nd edn*. (World Scientific Singapore, 2004).

7 Nelson, D. R. & Peliti, L. Fluctuations in membranes with crystalline and hexatic order. *Journal de Physique* **48**, 1085-1092, doi:10.1051/jphys:019870048070108500 (1987).

8 López-Polín, G. *et al.* Strain dependent elastic modulus of graphene. *arXiv:1504.05521 [cond-mat]* (2015).

9 Roldán, R., Fasolino, A., Zakharchenko, K. V. & Katsnelson, M. I. Suppression of anharmonicities in crystalline membranes by external strain. *Physical Review B* **83**, 174104, doi:10.1103/PhysRevB.83.174104 (2011).

10 Plimpton, S. Fast Parallel Algorithms for Short-Range Molecular Dynamics. *Journal of Computational Physics* **117**, 1-19 (1995).

11 Los, J. H. & Fasolino, A. Intrinsic long-range bond-order potential for carbon: Performance in Monte Carlo simulations of graphitization. *Phys. Rev. B* **68**, 024107 (2003).

12 Eckmann, A. *et al.* Probing the Nature of Defects in Graphene by Raman Spectroscopy. *Nano Lett.* **12**, 3925-3930, doi:10.1021/nl300901a (2012).

13 Cançado, L. G. *et al.* Quantifying Defects in Graphene via Raman Spectroscopy at Different Excitation Energies. *Nano Lett.* **11**, 3190-3196, doi:10.1021/nl201432g (2011).

14 Lehtinen, O., Kotakoski, J., Krasheninnikov, A. V. & Keinonen, J. Cutting and controlled modification of graphene with ion beams. *Nanotechnology* **22**, 175306, doi:10.1088/0957-4484/22/17/175306 (2011).
